# Does Wheat Genetically Modified for Disease Resistance Affect Root-Colonizing Pseudomonads and Arbuscular Mycorrhizal Fungi?

**DOI:** 10.1371/journal.pone.0053825

**Published:** 2013-01-23

**Authors:** Joana Beatrice Meyer, Yi Song-Wilson, Andrea Foetzki, Carolin Luginbühl, Michael Winzeler, Yvan Kneubühler, Caterina Matasci, Fabio Mascher-Frutschi, Olena Kalinina, Thomas Boller, Christoph Keel, Monika Maurhofer

**Affiliations:** 1 Plant Pathology, Institute of Integrative Biology, Swiss Federal Institute of Technology, Zurich, Switzerland; 2 Institute of Botany, University of Basel, Basel, Switzerland; 3 Agroscope Reckenholz-Tänikon Research Station ART, Zürich, Switzerland; 4 Agroscope Changins-Wädenswil Research Station ACW, Nyon, Switzerland; 5 Institute of Evolutionary Biology and Environmental Studies, University of Zurich, Zürich, Switzerland; 6 Department of Fundamental Microbiology, University of Lausanne, Lausanne, Switzerland; Nanjing Agricultural University, China

## Abstract

This study aimed to evaluate the impact of genetically modified (GM) wheat with introduced *pm3b* mildew resistance transgene, on two types of root-colonizing microorganisms, namely pseudomonads and arbuscular mycorrhizal fungi (AMF). Our investigations were carried out in field trials over three field seasons and at two locations. Serial dilution in selective King's B medium and microscopy were used to assess the abundance of cultivable pseudomonads and AMF, respectively. We developed a denaturing gradient gel electrophoresis (DGGE) method to characterize the diversity of the *pqqC* gene, which is involved in *Pseudomonas* phosphate solubilization. A major result was that in the first field season *Pseudomonas* abundances and diversity on roots of GM *pm3b* lines, but also on non-GM sister lines were different from those of the parental lines and conventional wheat cultivars. This indicates a strong effect of the procedures by which these plants were created, as GM and sister lines were generated via tissue cultures and propagated in the greenhouse. Moreover, *Pseudomonas* population sizes and DGGE profiles varied considerably between individual GM lines with different genomic locations of the *pm3b* transgene. At individual time points, differences in *Pseudomonas* and AMF accumulation between GM and control lines were detected, but they were not consistent and much less pronounced than differences detected between young and old plants, different conventional wheat cultivars or at different locations and field seasons. Thus, we conclude that impacts of GM wheat on plant-beneficial root-colonizing microorganisms are minor and not of ecological importance. The cultivation-independent *pqqC*-DGGE approach proved to be a useful tool for monitoring the dynamics of *Pseudomonas* populations in a wheat field and even sensitive enough for detecting population responses to altered plant physiology.

## Introduction

Plant-beneficial microorganisms are widely recognized as a crucial natural component of fertility in agricultural soils. Besides rhizobia and other N-fixing bacteria, there are two main groups of microorganisms known to be involved in plant growth promotion and plant health. The first group is the root-colonizing pseudomonads. These bacteria increase plant growth either directly by the production of phytohormones and other stimulants and by increasing the bioavailability of nutrients in the soil or indirectly by the suppression of plant diseases and the induction of systemic resistance in the plant [1]–[3]. One major problem in crop production is the limited bioavailability of the essential macroelement phosphorus (P), because it forms highly insoluble iron/aluminum oxide complexes in the soil [4]. Plant-beneficial pseudomonads are known to solubilize phosphate from these soil complexes, by the production of organic acids, mainly gluconic acid, for which they need the enzyme glucose dehydrogenase (GDH) and its cofactor pyrroloquinoline quinone (PQQ) [5], [6].

The second group of plant-beneficial microorganisms is arbuscular mycorrhizal fungi (AMF). These fungi of the phylum *Glomeromycota* form mutualistic symbioses with 80% of terrestrial plant families, facilitating the uptake of water and mineral nutrients to their host plants while receiving carbohydrates in exchange [7]. They can also increase the plants' resistance to biotic and abiotic stress factors while improving soil stability [8]. Because of their unequivocal importance, AMF are considered an excellent indicator of possible ecological impacts of genetically modified (GM) crops containing antifungal transgenes on soil microbial communities [9].

In wheat production, diseases caused by fungal pathogens represent a major problem. Genetically modified (GM) wheat engineered for pathogen resistance might represent a valuable cost efficient and ecological alternative to the large use of fungicides. The field application of GM plants, however, might have undesirable consequences on the surrounding ecosystem including plant-beneficial soil microorganisms. Non-target organisms could be affected either by the product of the transgene itself or indirectly by interaction with an altered plant phenotype [10]–[13].

The impacts of transgenes on soil microorganisms have mainly been assessed with GM oilseed rape, maize, potatoes and tobacco [14]–[21]. The majority of studies on GM impact monitored changes in diversity of whole rhizosphere-associated fungal and bacterial communities and revealed minor or no effect of the GM plant on the studied microorganisms. Although some of the work performed so far included the genus *Pseudomonas* [16], [17], [19], up to now there is no knowledge on the impact of wheat engineered for fungal resistance on plant-beneficial P-solubilizing pseudomonads and arbuscular mycorrhizal fungi which are relevant indicators of microorganism-derived soil fertility.

In this study, which is part of comprehensive field investigations on GM wheat by a consortium of different research groups (wheat consortium; part of the National Research Program NRP 59, Available: http://www.NRP59.ch, Accessed 2012 Dec 13), we aimed at filling this gap, by investigating the impact of GM wheat lines carrying introduced fungal resistance transgenes on P-solubilizing pseudomonads and AMF. To investigate transgene impact, we used as control lines the non-GM sister lines which are the descendants from the same primary transformants (T_0_ plants) as the GM lines. The non-GM sister lines lack both the transgene and the selectable marker gene cassette (null segregant lines), as shown by Southern blot analysis [10], but they had gone through exactly the same tissue culture and regeneration process as the GM lines, and thus might carry the same (epi)genetic or phenotypic alterations. Potential effects of transgenes introduced into wheat on accumulation and diversity of root-colonizing pseudomonads and AMF were put in relation to variations found between different wheat cultivars or effects caused by factors such as the plant age, fertilizer addition, disease pressure, field season and field location.

The following tasks were envisaged: i) a comparison of the ability of GM and non-GM wheat lines to accumulate and sustain root-colonizing pseudomonads and AMF and ii) a monitoring of the genetic diversity of phosphate-solubilizing pseudomonads on the roots of GM wheat lines and their non-GM parental/sister lines. To the latter purpose, a denaturing gradient gel electrophoresis (DGGE) technique was developed for analyzing the diversity of the PQQ biosynthetic gene *pqqC*, a gene we have shown in a previous study to be a good molecular marker for investigations on natural populations of P-solubilizing pseudomonads [6].

## Results

### Novel *pqqC*-DGGE fingerprinting technique

#### *pqqC*-DGGE fingerprinting for *Pseudomonas* test strains

We have developed a PCR-DGGE method to characterize the diversity of the *pqqC* gene within the genus *Pseudomonas*. The suitability of this method was tested by analyzing a set of 60 pseudomonads listed in Table S1. Twelve different *pqqC*-DGGE bands were found for the tested bacteria. Sequences of these bands were integrated into a *Pseudomonas* phylogenetic tree shown in Fig. S1.

#### Diversity of *pqqC* in *Pseudomonas* populations on wheat roots

The diversity of pseudomonads was assessed in the rhizosphere of GM and non-GM wheat by *pqqC*-DGGE in field trials performed at Reckenholz in the years 2008 and 2009. In total, 34 bands with different migration in the gels were found. Bands that migrated to the same position were designated with the same letter. Twelve main bands (bands A to N) were included into the analysis. Main bands were directly sequenced or cloned and sequenced (sequences were submitted to GenBank under the accession numbers JX861212 - 230) and then compared to published *pqqC* sequences. The placement of the DGGE band sequences in a *Pseudomonas* phylogenetic tree is shown in Fig. S1. The majority of sequences of seven main bands (A, D, E, K, M and N), including band C, the most prominent band which was present in all samples could be assigned to the *P. fluorescens* phylogenetic subgroup 1 g as defined by Meyer et al. [6]. Bands G and I clustered with *P. fluorescens* subgroup 1b, bands H and J could be assigned to *P. fluorescens* subgroup 1e, band F to *P. syringae*, and some cloned sequences of band M obtained from samples of 2009 to *P. fluorescens* subgroup 1b.

### Impacts on *Pseudomonas* abundance and diversity

#### Specificity of KB+++ MPN technique for pseudomonads

Amplification of the 16S rRNA gene with primers specific for the genus *Pseudomonas* from bacteria grown in microtiter plates (samples from Reckenholz trial 2008) revealed that 97–98% of the root bacteria grown in KBM+++ are pseudomonads with no differences between individual wheat lines or cultivars. We considered this small fraction of non-pseudomonads as negligible and therefore use in this study the term cultivable pseudomonads for the bacteria enumerated by this method.

#### Impact of plant production procedures

**Figure 1:**
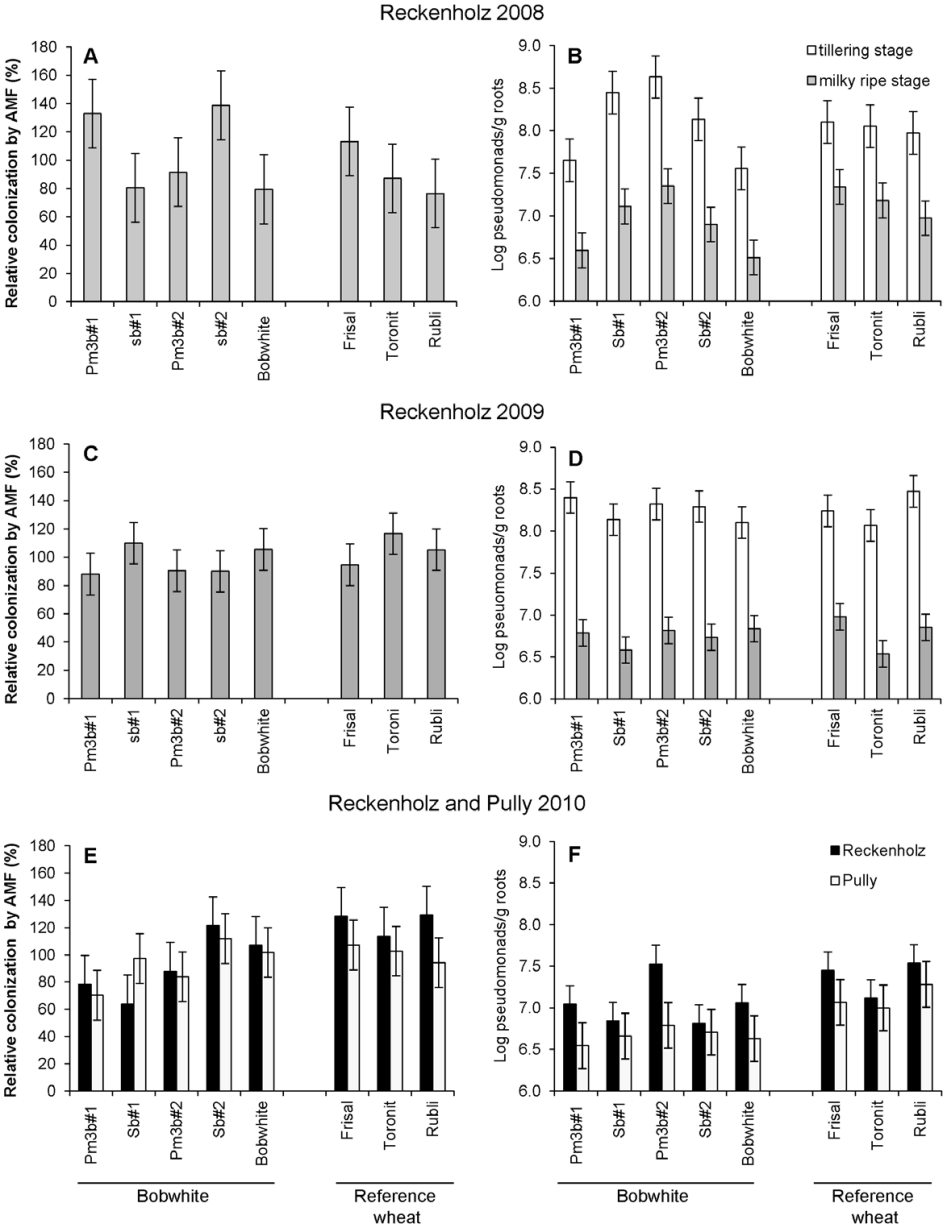
Colonization of roots of transgenic and non-transgenic wheat by arbuscular mycorrhizal fungi (AMF) (A, C, E) and cultivable pseudomonads (B, D, F) in field trials performed in 2008, 2009 and 2010. Roots were sampled at the tillering (B, D) and the milky ripe stage (A–F) at Reckenholz (A–F) and Pully (E, F). Relative colonization: root colonization by AMF was expressed for each wheat line/cultivar relative to the average colonization calculated for all wheat lines. Significant differences (P<0.05) are listed in Table 1. Pm3b = GM lines, Sb = non-GM sister lines. Bars indicate the standard error of the estimate. Significant differences (P<0.05) detected by pairwise comparisons A) Pm3b#1 vs Sb#1; B) tillering stage: Pm3b#1 vs Sb#1 and Pm3b#2; B) milky ripe stage: Pm3b#1 vs Sb#1 and Pm3b#2, Sb#1 vs Pm3b#2, Bobwhite vs Frisal and Toronit; D) milky ripe stage: Toronit vs Frisal; F) Reckenholz: Sb#2 vs Pm3b#2; F) Pully: Bobwhite vs Rubli.

**Figure 2:**
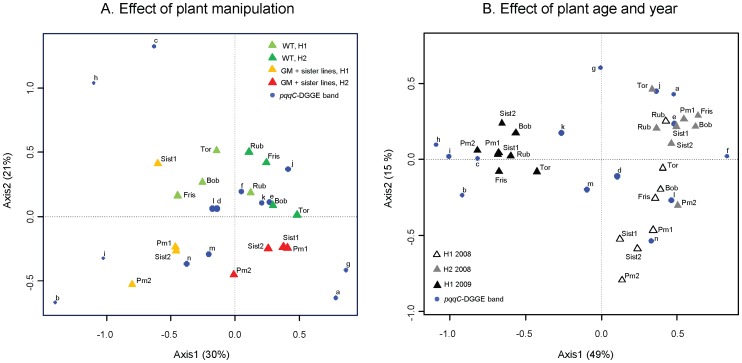
Ordination biplots generated by correspondence analysis (CA) of *pqqC*-DGGE profiles from wheat root samples. DGGE bands (letters a–n) incorporated in the CA are shown. The size and position of the blue bullets reflect the contribution of each band to the distribution pattern. Graph A shows plants grown in the Reckenholz field trial in 2008 (experiment 1) and sampled at the tillering (H1) and at the milky ripe stage (H2). Graph B additionally includes data from Reckenholz field trial 2009 obtained from plants harvested at the tillering stage (H1). GM and SIST = GM Bobwhite (*pm3b*) and non-GM Bobwhite sister lines; WT = conventional non-GM wheat cultivars Frisal (or Fris), Toronit (or Tor), Rubli (or Rub) and Bobwhite (or Bob).

**Figure 3:**
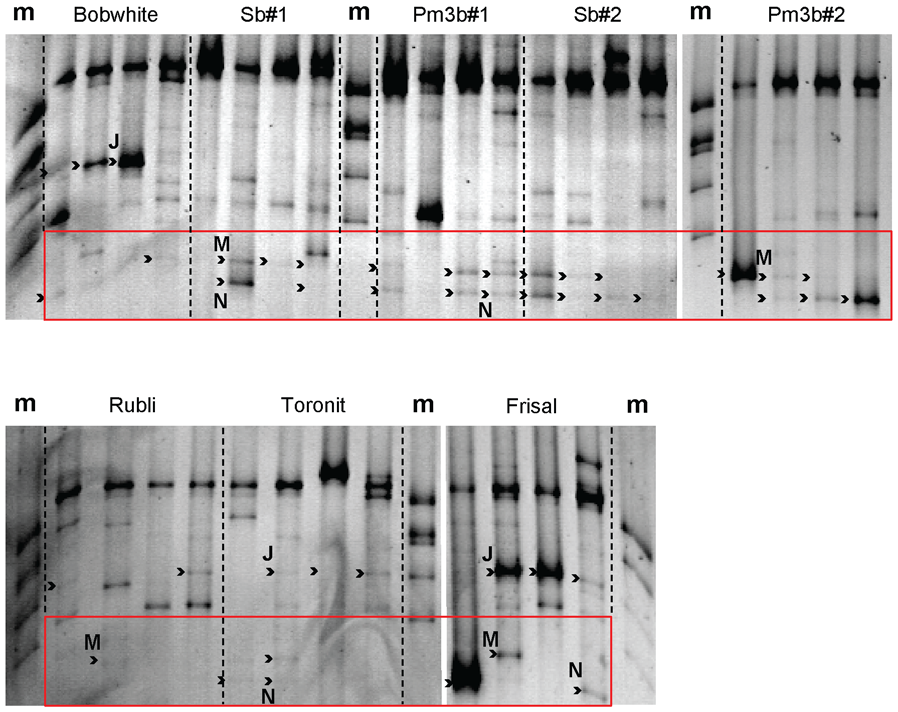
Impact of plant production procedures on DGGE profiles. *Pseudomonas*-specific *pqqC* DGGE was performed with DNA extracted from roots of wheat at the tillering stage grown in the Reckenholz field trial 2008. The four lanes derived from four replicate field plots per line/cultivar are shown. Arrows indicate the *pqqC* DGGE band J, which is enriched on root profiles of conventional wheat cultivars, and bands M and N which are enriched on root profiles of the Bobwhite GM and sister lines. These bands correspond to *P. fluorescens* subgroup 1e (band J) and *P. fluorescens* subgroup 1 g (band M and N), as defined by Meyer et al. [6]. Pm3b#1 and Pm3b#2 are the GM lines with introduced *pm3b* transgene, Sb#1 and Sb#2 are their respective non-GM sister lines and Bobwhite is the parental line. Frisal, Toronit and Rubli are conventional wheat cultivars. Bobwhite GM and sister lines originate from T_0_ GM plants regenerated from tissue cultures, sister lines lost the transgene in the T_1_ generation due to segregation. Seeds of GM and sister lines used in the field trial were produced in the greenhouse, seeds of Bobwhite, Frisal, Toronit and Rubli were produced in the field. m, DGGE standard marker, consisting of *pqqC* fragments of *Pseudomonas* strains (from the upper to the lower band): *Pseudomonas chlororaphis* LMG1245, *P. fluorescens* Q2-87, *P. fluorescens* CHA0, *P. fluorescens* K94.37, *P. corrugata* LMG2172.

**Figure 4:**
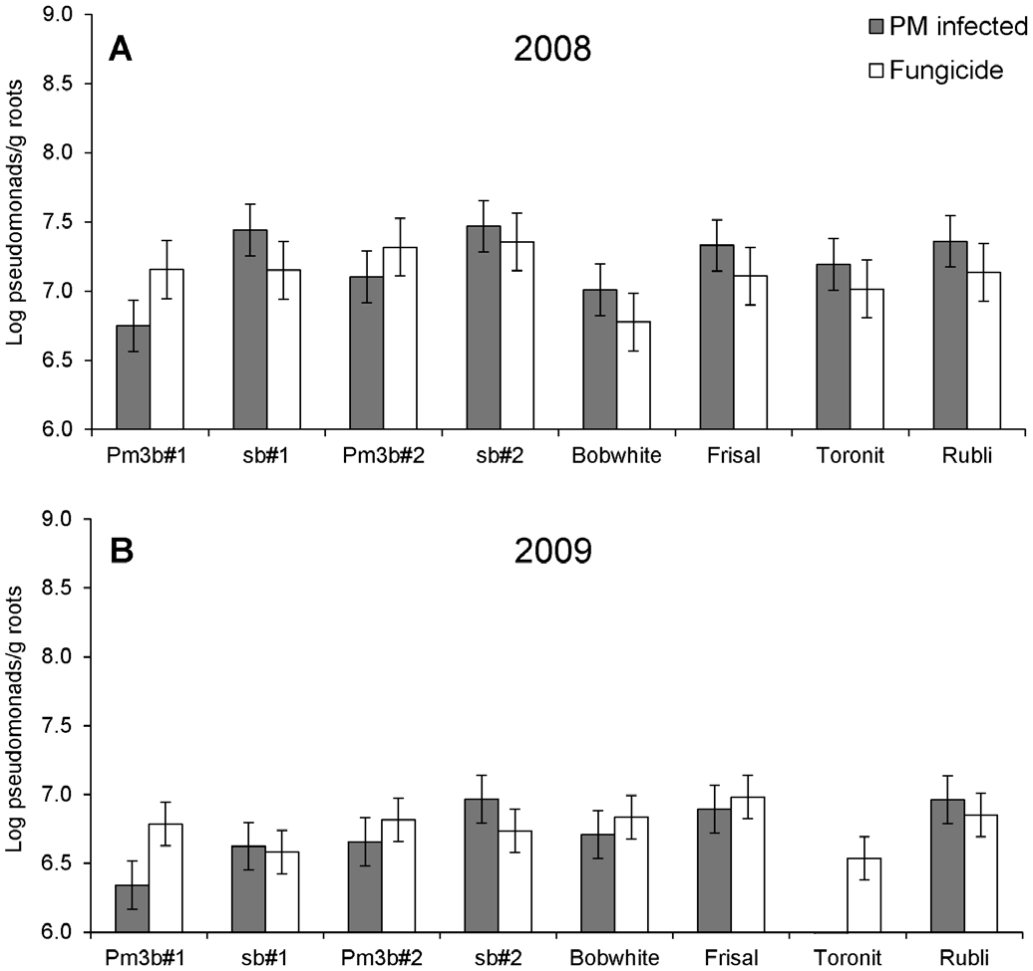
Impact of powdery mildew infection on population sizes of cultivable pseudomonads on roots of GM and non-GM wheat grown in field trials at Reckenholz 2008 (experiment 2) (A) and 2009 (B). Roots were sampled at the milky ripe stage. PM infection = plants inoculated with powdery mildew. Fungicide = plants were treated with the fungicide Prosper. In 2009 data for the powdery mildew infected Toronit are missing. Bars indicate standard errors of the means. Mildew-infected plants differed significantly form fungicide treated plants in 2008, but not in 2009 (Table 1). Significant differences (P<0.05) detected by pairwise comparisons: A) PM infected: Pm3b#1 vs Sb#1, B) Fungicide: Toronit vs Frisal.

A particularly interesting result of our study was the impact of the manner plant lines were produced. In the Reckenholz trial 2008, we found for both *Pseudomonas* diversity and population sizes significant differences between the Bobwhite parental cultivar and the corresponding non-GM sister and *pm3b* lines which, in contrast to the all conventional cultivars, were generated from tissue culture and propagated in the greenhouse. Especially in the first field season, *Pseudomonas* population sizes on the parental Bobwhite cultivar were significantly smaller than on *pm3b* and sister lines (Table 1, Fig. 1B). Regarding diversity, the generalized linear model (glm) analysis showed an impact of the factor plant production procedure in 2008 (P<0.1), but not in 2009, indicating that GM Bobwhite sister/lines were colonized by more *pqqC* genotypes than the parental/conventional cultivars (Table S2). In the correspondence analysis (CA) performed on frequency matrices (pooled results of four replicates) of DGGE profiles, two “clouds” of data points are visualized, separated along the second axis (21% of the total variance), one containing mainly the conventional wheat cultivars, the other the GM/sister lines (Fig. 2A). This separation is also visible on a MDS plot performed on binary matrices (presence/absence of genotypes for each plant replicate) (graph A in Fig. S2). MRPP analysis on both binary and frequency data sets resulted in a significant difference (P<0.05) between these two plants groups, the conventional cultivars versus the GM and sister lines for the plants harvested in 2008 at the tillering stage (binary data: A = 0.12, P = 0.012, frequency data: A = 0.21, P = 0.023) and at the milky ripe stage (binary data: A = 0.15, P<0.01; frequency data: A = 0.17, P = 0.024). The differences between the two groups were that the conventional cultivars, but not the lines generated in the laboratory (GM and sister lines) accumulated genotype (band) J (belonging to *P. fluorescens* subgroup 1e) (Fig. 2A and Fig. 3) and that the manipulated lines strongly accumulated genotypes M and N (both belonging to *P. fluorescens* subgroup 1 g), which were much less abundant on the control cultivars (Fig. 2A and Fig. 3). In 2009 at the tillering stage, the differences between lines were smaller and conventional cultivars did no significantly differ from the GM/sister lines as found with MRPP (MRPP for binary data: A = 0.020, P = 0.058 and frequency data: A = 0.030, P = 0.14). Regarding population sizes, the differences also disappeared in 2009 and 2010 (Fig. 1D and 1F, Fig. 4).

**Table 1:** Significant interactions (P≤0.05) found in the field trials at Reckenholz and Pully (GenStat 13^th^ edition VSN International).

Analyzed samples	Significant effects	
	Total pseudomonads per g roots^b^	Total AMF per g roots
**Reckenholz 2008 - experiment 1** a		
**Plant development trial**		
**Both plant ages together**	Plant age	Plant age
	(old plants<young plants)	(young plants<old plants)
Tillering stage	Plant production procedures	-
	(Bobwhite<GM and sister lines)	
	Transgene position	
	(*Pm3b#1<Pm3b#2*)^c^	
	*pm3b* transgene	
	(*Pm3b#1<Sb#1*)	
Milky ripe stage	Wheat cultivar	-
	(*Bobwhite<Frisal, Toronit*)	
	Plant production procedures	
	(Bobwhite<GM and sister lines)	
	Transgene position	
	(*Pm3b#1<Pm3b#2*)	
	*pm3b* transgene	*pm3b* transgene
	(*Pm3b#1<Sb#*1 and *Sb#2<Pm3b#2*)	(*Sb#1<Pm3b#1*)
**Reckenholz 2008 - experiment 2** a		
**Powdery mildew trial**		
**Both treatments together, milky ripe stage**	Fungicide/mildew (fungicide treated plants<mildew infected plants)	*pm3b* transgene
	Plant production procedures	
	(Bobwhite<GM and sister lines)	
	Mildew infection×GM vs sister lines	
Powdery mildew treatment	*pm3b* transgene	Transgene position
	(GM lines<sister lines)	(*Pm3b#2<Pm3b#1*)
		*pm3b* transgene
		(*Pm3b#2<Sb#2*)
Fungicide treatment	-	Transgene position
		(*Pm3b#2<Pm3b#1*)
		*pm3b* transgene
		(*Pm3b#2<Sb#2*)
**Reckenholz 2009**		
**Both plant ages and both treatments**		
**together**	Plant age	Plant age
	(old plants<young plants)	(young plants<old plants)
	Wheat cultivar	Wheat cultivar
Tillering stage, fungicide treatment	-	-
Tillering stage, powdery mildew treatment	Wheat cultivar	-
	(*Bobwhite, Frisal, Toronit<Rubli*)	
Milky ripe stage, fungicide treatment	Wheat cultivar	-
	(*Toronit<Frisal*)	
Milky ripe stage, powdery mildew treatment	-	Wheat cultivar
		(*Bobwhite<Toronit, Rubli*)
**Reckenholz and Pully 2010**		
**Both field sites together**	Field site (Pully<Reckenholz)	-
Reckenholz, milky ripe stage	*pm3b* transgene	-
	(*Sb#2<Pm3b#2*)	
Pully, milky ripe stage	Wheat cultivar	-
	(*Bobwhite<Rubli*)	

a In 2008 two experiments were performed: experiment 1 with comparisons of different plant lines at two development stages and experiment 2 with comparisons of different plant lines grown in mildew-infected and in fungicide-treated plots at the milky ripe stage. Plants were artificially infected by planting heavily mildew-infected plants in spreader rows [10].

b Cultivable pseudomonads were quantified by serial dilution in KB+++ medium.

c Significant differences resulting from pairwise comparisons of individual wheat lines/cultivars are indicated in italics. eg. *Toronit<Frisal* = Toronit had significantly smaller *Pseudomonas* population sizes on its roots (or root surface colonized by AMF) compared to Frisal.

#### Impact of introduced disease resistance genes/transgene position effects

For the two Bobwhite lines transformed with the powdery-mildew resistance gene *pm3b*, significant differences to the corresponding control sister lines were found at certain time points, but varied depending on the GM line. At the Reckenholz site in 2008, line Pm3b#1 accumulated at both development stages significantly less pseudomonads than its corresponding sister line Sb#1 (Fig. 1B). In contrast, Pm3b#2 accumulated significantly more pseudomonads than Sb#2 in the Reckenholz trials 2008 and 2010 (Fig. 1B and 1F). In the Reckenholz trial 2009 and the Pully trial 2010 no *pm3b* transgene effect was found (Fig. 1D and 1F). Interestingly *pm3b* lines behaved differently compared to their sister lines in the Reckenholz mildew treatment in 2008 and accumulated less pseudomonads in plots infected with the pathogen in comparison to fungicide-treated plots. In contrast, their sister lines and the conventional cultivars accumulated more pseudomonads in mildew-infected plots (Table 1, Fig. 4). The same difference between GM and sister lines was observed again in the Reckenholz trial 2009 (Fig. 4). Throughout the experiments we observed a transgene position effect; Pm3b#2 accumulated in most comparisons more pseudomonads than Pm3b#1. This difference was highly significant in 2008 (Table 1, Fig. 1B).

#### Major impacts: plant age, cultivar, cropping season and field site

The factors which had clearly the strongest impact on size and diversity of *Pseudomonas* populations on wheat roots were the plant age, the cropping season, and regarding population sizes also the cultivar (Table 1, Fig. 1, Fig. 2 and Fig. S2). In the majority of the field trials we observed a consistent cultivar impact with Bobwhite and Toronit accumulating less pseudomonads than Frisal and Rubli (Table 1, Fig. 1 and Fig. 4). The most pronounced impact on *Pseudomonas* populations, however, was the plant age. In the years 2008 and 2009 at the Reckenholz location, *Pseudomonas* populations decreased drastically (3–15 times in 2008, 10–45 times in 2009), from roots of young plants to roots of older plants (Fig. 1B and 1D). The average root colonization at the tillering stage calculated for all wheat cultivars/lines was log 8.1 (2008) and log 8.3 (2009), and at the milky ripe stage log 7.0 (2008) and log 6.8 (2009). The plant age also significantly impacted on *Pseudomonas* diversity in 2008 and 2009 as higher numbers of *pqqC* genotypes were detected at the milky ripe stage (Table S2).

Fig. 2 shows correspondence analyses (CAs) of band frequency matrices summarizing the factors impacting most on *pqqC*-DGGE patterns in the field. Fig. 2A shows data from 2008 only. The first three axes of the CA, which account for most of the variability, explained 65.5% of the total variance. Plant age significantly separates data into two groups (MRPP analysis: A = 0.090, P = 0.003), with a clear distinction on the first axis (30% of the total variance). In Fig. 2B including data from 2009, the first three axes of the CA explained 76.6% of the total variance. This figure shows that the field season even had the stronger effect (MRPP analysis: A = 0.25, P<0.01) than the plant age with a clear separation of the DGGE patterns on the first axis explaining 49% of the total variance. Mostly responsible for differences between seasons were genotypes H and I (H: *P. fluorescens* subgroup 1e; I: P. *fluorescens* subgroup 1b) which were present on all roots in 2009 but much less frequent in 2008. MDS analyses based on band presence/absence data of individual replicates revealed similar results also identifying the field season (MRPP analysis: A = 0.20, P<0.01) and the plant age (MRPP analysis: A = 0.10, P<0.01) as the factors impacting significantly on *pqqC* DGGE patterns (Fig. S2).

Also the glm analysis identified the field season as a factor impacting on *pqqC* diversity, as more *pqqC* genotypes were found in 2009 (Table S2) compared to 2008. Differences among field sites were analyzed in 2010 only for population sizes and not for *pqqC* diversity. *Pseudomonas* populations were lower at the Pully site than at the Reckenholz site (Table 1, Fig. 1F).

### Impacts on AMF abundance

Only few of the investigated factors had a significant impact on AMF root populations. The plant age clearly had the strongest effect on populations of wheat root-colonizing AMF (Table 1). In contrast to the pseudomonads, AMF populations markedly increased (1.5–3 times) from young to older plants and reached 23–26% root surface coverage at the milky ripe stage.

In 2008 a significant transgene impact was found in both experiments (Table 1). In experiment 1 root colonization of Pm3b#1, was colonized to a 65% higher extent than the corresponding sister line (Fig. 1A). For Pm3b#2, however, opposite effects were found with the GM line displaying lower AMF colonization rates than the sister line. This effect was only significant in the mildew infection experiment (exp. 2). In both treatments (fungicide and mildew) the colonization of Pm3b#2 roots was 2.5 times lower compared to the non-transgenic sister line Sb#2 and AMF covered only 12% of the root surface.

In contrast to the pseudomonads, for AMF only in 2009 in the mildew treatment a significant cultivar effect was observed (Table 1). The AMF-covered root surface of Toronit and Rubli (35%) was 50% larger than that of Bobwhite, which only reached 23%.

## Discussion

We report here on a large-scale field study which aimed at comparing the impact of GM wheat modified for disease resistance on plant-beneficial pseudomonads and AMF with the impact of other factors root-colonizing microorganisms encountered in agricultural systems. One of the major outcomes was that not the transgene insertion did alter microbial diversity and population sizes, but that the procedure of producing GM wheat itself showed a strong influence. Both sister lines and *pm3b* Bobwhite lines supported communities of pseudomonads that differed in their composition from those of the parental lines, but also from the other tested conventional cultivars (Table S2, Fig. 2A). Furthermore, in the first field season the parental Bobwhite cultivar accumulated less pseudomonads than the GM and sister lines (Table 1, Fig. 1B). This demonstrates the importance of using the correct control lines. If the Bobwhite sister lines (null segregants) had been omitted in the field experiments and only the parental line had been used as control as in many earlier studies on GM plants, differences found between GM and non-GM parental lines most probably would have been attributed to effects of the introduced disease resistance genes. Most transgene impact studies, so far, included as control mainly the non-transgenic parental line and only few studies also included other controls such as lines carrying only the selectable marker, lines carrying the empty vector or non-transgenic isolines [22]–[27]. Lines carrying only the selectable marker or the empty vector, however, are produced independently from the GM lines, whereas each sister line we used here derives from the same F1 generation as the corresponding GM line. The differentiation GM/sister Bobwhite lines and conventional wheat disappeared in the subsequent years where field propagated seeds had been used for all entries in the field trials (Table 1 and S2, Fig. 1). We suggest that these lines had quickly adapted to field conditions.

The major differences between conventional cultivars (including Bobwhite) and the Bobwhite GM and sister lines were the preference of the manipulated lines for genotypes M and N identified as belonging to *P. fluorescens* subgroup 1 g containing also reference strain SBW25 (Fig. S1) [6] and of the conventional wheat for genotype J showing most similarity to *P. fluorescens* subgroup 1e. Interestingly genotype M was also strongly induced by powdery mildew infection and mechanical injury by vandals (data not shown). Plants, which accumulated genotype M, in general also supported higher *Pseudomonas* populations. It would be interesting to assess in further experiments whether certain *Pseudomonas* genotypes are particularly adapted to root exudates released by stressed plants and are able to outcompete others under such conditions.

What exactly had caused these differences in *Pseudomonas* abundance and diversity between Bobwhite sister/GM lines and the parental line remains unclear. Since Bobwhite GM and sister lines did not differ in development [10], we can exclude that the detected differences in *Pseudomonas* communities were due to differences in plant development stages at the time of sampling. Obviously the manner the seeds of these plants used for the Reckenholz trial 2008 were produced had altered plant physiology. GM and sister lines underwent the transformation process, were generated via tissue cultures and then propagated in the greenhouse. Any of these procedures, alone or in combination, might have had somaclonal effects resulting in physiological changes as shown previously [28], [29]. Altered plant physiology due to stress, pathogen attack, genotype or environment effects can alter the amount and/or the composition of root exudates, which can have a strong impact on root-colonizing pseudomonads and their expression of plant-beneficial traits [5], [30]–[33]. Another interesting outcome was that changed plant physiologies caused by different genomic locations of the *pm3b* transgene caused similar differences as found between transgenic/sister lines and conventional wheat. In 2008 where most pronounced differences between GM and sister lines were found, Pm3b#1 accumulated less pseudomonads but more AMF, Pm3b#2 in contrast, more pseudomonads but less AMF than the corresponding sister line (Fig. 1A and 1B). In addition more *pqqC* genotypes were found on Pm3b#1 compared to Pm3b#2. So what is the difference between these lines? Pm3b#1 expresses the transgene at much lower levels than Pm3b#2 [10] and does not display strong pleiotropic effects. In contrast, Pm3b#2 had the highest *pm3b* expression levels, which resulted in several pleiotropic effects such as chlorotic leaves, reduced stomatal conductance, smaller plant height and altered flower morphology [10] what caused the side-effect of infection by the ergot pathogen *Claviceps purpurea* [13]. These physiological differences might have resulted in altered root exudations, which in the end had an impact on microbial abundances. A study illustrating such an effect is that of Li et al. [34] which shows that GM cotton lines transformed with Cry1Ac/CpTI- and Cry1Ac, release root exudates with increased sugar content what causes them to be more susceptible to *Fusarium oxysporum* infection than the parental line.

In contrast to the GM impact, the cultivar impact on *Pseudomonas* accumulation was very consistent throughout the experiments as for example the Mexican cultivar Bobwhite supported generally lower populations than the Swiss cultivars Frisal and Rubli over three field seasons, two field locations and two treatments. The wheat genotype is indeed known to modulate root-associated pseudomonads and their activity through the release of specific compounds in the root exudate [31], [32], [35], [36]. AMF populations, however, were much less affected by the wheat cultivar.

Finally, the major factors that affected AMF and *Pseudomonas* accumulation and also *Pseudomonas* diversity were the plant age, the field site and the field season. Considering plant age, *Pseudomonas* population sizes on the roots of older plants were considerably lower than at the tillering stage (Fig. 1B and 1D). In contrast, the area of roots colonized by AMF increased from young to older plants, as is typical for this symbiosis. Pseudomonads are considered to be aggressive colonizers, adapted to rapidly colonizing free niches, such as the roots of young plants rich in carbon sources and amino acids and outcompeting other microorganisms [37]. On older plants, population sizes decrease since other microorganisms, among them also AMF, establish themselves in the rhizosphere. The decline from young to mature plants has been described earlier [38], [39]. Opposite dynamics of AMF and *Pseudomonas* populations in wheat fields in response to the plant age have also been observed before [38]. It was shown in a field experiment that inoculation of spring wheat with different *Pseudomonas* spp. can reduce AMF colonization [40]. Pseudomonads have also been found to have a direct inhibitory effect on AMF spore germination through the release of volatile compounds [41]. It is therefore difficult to distinguish between physiological effects of a growing plant on *Pseudomonas* and AMF population sizes and possible effects of direct interaction of the two groups of microorganisms. Beside plant-induced changes, different environmental conditions (e.g., soil properties, weather conditions) occurring at different field sites and in different cropping seasons considerably shaped microorganism communities in our study, a common phenomenon, which is especially observed for the very dynamic pseudomonads since these are sensitive to many environmental factors [35], [42]–[48].

In summary, comparing all studied factors, introduced transgenes had only a minor effect on the microbial populations. Our study on AMF and pseudomonads revealed similar results as studies on other non-target organisms performed by different groups in the frame of the same field experiments. These studies showed no relevant impact of the *pm3b* on the abundance of aphids, the cereal leaf beetle *Oulema malanopus* or the wheat stem fly *Chlorops pumilionis* [49], on the dipterans *Drosophila melanogaster* and *Megaselia scalaris* [50], on the annelid *Enchytraeus albidus* [51], on aphid-parasitoid food webs [52], [53] and on soil decomposition activity and soil fauna [54].

Work on the impact of GM crop plants on target and non-target fungi and on microbial communities has been reviewed by several authors [14], [15], [18], [20], [21]. As summarized in these reviews, most studies on GM plants had little or no impact on non-target fungi and bacteria. However, in some exceptional cases, transgenic plants showed a reduction of colonization by AMF [55]. In the extensive review by Stefani and Hamelin [18] it is concluded that transgenic plants should be evaluated on a case-by-case basis. Our results indicate that the transgenic wheat lines studied are not affected with regard to their ability to form the arbuscular mycorrhizal symbiosis and to be colonized by pseudomonads.

### 

#### Conclusion

Our field study is the first evaluating the impact of GM wheat on two specific groups of root-colonizing microorganisms over three consecutive field seasons and at two different field sites. The greatest advantage was the possibility to compare each GM line with the corresponding T_0_ segregant sister line, which allowed distinguishing between true transgene effects and effects of the methods by which GM lines were produced. Effectively, we show that such effects can be very relevant and if not considered might lead to false conclusions about GM impacts. The detected transgene effects were minor, opposite for individual GM lines and not consistent; we thus conclude that they are not of ecological importance. We furthermore demonstrated that *pqqC* is a molecular marker sensitive enough to detect differences even between *Pseudomonas* community compositions on plants grown from seeds produced in the field and on plants grown from seeds derived from laboratory and greenhouse production.

## Materials and Methods

### Bacteria and plants used in the study

#### Pseudomonas strains

*Pseudomonas* spp. strains used in this study are listed in Table S1. Test isolates RW09-C35 to C65 for DGGE (see below) were isolated from the roots of wheat grown in the Reckenholz field trial 2008 as described earlier [6]. Bacterial strains were grown at 27°C in 10 ml liquid King's medium B [56] agar containing 40 µg/ml ampicillin, 13 µg/ml chloramphenicol and 100 µg/ml cycloheximide (KBM +++) for 24 h on a rotary shaker (Kühner AG, Birsfelden, Switzerland) at 120 rpm. For DGGE, genomic DNA from bacterial strains used as template in PCR was obtained by lysing bacterial suspensions for 10 min at 96°C, subsequent centrifugation and collection of the supernatants.

#### Wheat cultivars

The conventional wheat cultivars used for investigation of *Pseudomonas* and AMF populations on roots were the Swiss cultivars Toronit, Rubli and Frisal and the Mexican cultivar Bobwhite SH 98 26. Two GM Bobwhite lines Pm3b#1 and Pm3b#2 used in the field trials were created by inserting the *pm3b* gene of hexaploid wheat into the genome of the parental line Bobwhite [10], [13]. *pm3b* confers race-specific disease resistance against powdery mildew. The two GM lines carry the transgene at a different position in the genome. For each GM line the correspondent null segregant line (lines Sb#1 and Sb#2, here called sister lines), which did neither inherit the transgene nor the selectable marker was used as control line in the experiments [10].

### Field experiments 2008–2010

Field experiments were performed in Switzerland at the research stations Agroscope Reckenholz-Tänikon (ART), location Reckenholz, Zürich in 2008, 2009 and 2010 and Agroscope Changins-Wädenswil (ACW), location Pully in 2010 within the frame of the National Research Program NRP59 founded by the Swiss National Science Foundation. Field experiments consisted of four (2008, 2010) or five (2009) replicate blocks with one plot per plant line and block. Plot widths ranged from 1.1 to 1.3 m and plot lengths from 1.3 m (ART 2008 experiment 2, ART 2009), 4 m (ART 2010) 4.5 m (ACW 2010) to 7 m (ART 2008, experiment 1). In order to achieve comparable samples between years, samples have always been taken in the two middle rows (regarding plot width) and 60 cm from the plot edge, independently of the plot length. Nitrogen fertilizer was applied as NH_4_NO_3_. Nitrogen, phosphorus and potassium were applied in Reckenholz fields as a total of 110 kg N ha^−1^, 46 kg P_2_O_5_ ha^−1^ and 60 kg K_2_O ha^−1^ and in Pully as a total of 80 kg N ha^−1^, 50 kg P_2_O_5_ ha^−1^ and 100 kg K_2_O ha^−1^. In 2008 (only experiment 2) and 2009, an additional treatment was made, where plants were artificially infected with powdery mildew as described by Brunner et al. [10]. In the Reckenholz trials of 2008 and 2009, a first sampling was performed when plants were at the tillering stage (BBCH stage 22–25) [57] and a second sampling when plants were at the milky ripe stage (BBCH 73–77). In Reckenholz and Pully trials 2010, plants were only sampled at the milky ripe stage. Ten plants per plot were dug out and their roots were pooled. Thus, for each field trial four (five in the Reckenholz trial 2009) samples per plant line (one pooled sample per replicate) were analyzed. The root-adhering soil was gently removed. Root samples were then rinsed on a sieve (mesh size 1 cm) with water to wash away remaining soil particles and subsequently shortly dried on paper tissues. Roots of plants sampled in one plot were weighed and placed in a 100-ml Erlenmeyer flask containing 50 ml sterile 0.9% NaCl solution. Samples were shaken for 30 min on a Mini-shaker (Kühner AG, Birsfelden, Switzerland) at 350 rpm and from each replicate 20 µl of the root suspension were taken for determination of population sizes. The remaining root suspension including the roots was stored at −20°C and later used for DNA extraction.

### Determination of cultivable *Pseudomonas* population sizes on roots

From each root suspension prepared as described above 20 µl were serially diluted (1∶10) in 96-well microtiter plates (Greiner Bio-one GmbH, Frickenhausen, Germany) part-filled with 180 µl *Pseudomonas* selective KBM +++ medium. Microtiter plates were incubated under slight agitation at 27°C in the dark. Bacterial growth was assessed after 48 h and total *P. fluorescens* cell numbers per gram root fresh weight were calculated using “Most Probable Number” (MPN) calculations as described by Svercel et al. [46].

#### Determination of specificity of KBM +++ medium

Since the KBM +++ medium is not strictly *Pseudomonas*-specific we performed a *Pseudomonas*-specific 16S rRNA-PCR on the samples of the year 2008 in order to determine the percentage of non-pseudomonads present on wheat roots which are able to grow in this medium. The proportion of rhizosphere pseudomonads carrying the 16S rRNA gene was examined using the MPN-PCR approach described by Svercel et al. [46]. Briefly, from each dilution in the microtiter plates 2 µl of heat-lyzed bacterial suspensions were used as templates for amplification of 16S rRNA using the *Pseudomonas*-specific 16S rRNA primers described by Widmer et al. [58] and the same reaction mix and the same PCR conditions as described below for *pqqC* PCR.

### Quantification of root colonization by arbuscular mycorrhizal fungi (AMF)

Root samples were kept in 50% ethanol and stained with 0.05% Trypan blue as previously described [59]. The stained roots were cut into 1–1.5 cm pieces, mounted on glass slides and observed under a microscope at 200× magnification (Zeiss Axioplan D-7082, Oberkochen, Germany). These samples were then analyzed using the standard magnified intersections method to quantify AMF colonization in the roots [60] but instead of counting 100 intersections per slide (standard), we increased the test to 150 intersections to be more precise. To avoid any subjectivity in the staining and counting procedures, we used a double-blind procedure for all the samples reported in this paper.

### *pqqC* based diversity of wheat root colonizing pseudomonads

#### DNA extraction and PCR conditions

50 ml of root suspensions (prepared as described above) were centrifuged for 10 min at 3600 rpm. The total DNA was then extracted from the resulting pellet and from root pieces (0.5 g) using the Fast DNA Spin Kit for soil (MP Biomedicals, Irvine, CA, USA) as described [6]. Amplifications of *pqqC* in bacterial lysates were carried out in 20-µl reaction mixtures containing 1× ThermoPol Buffer (New England Biolabs, Inc., Beverly, MA, USA), 100 µM of each dNTP, 0.4 µM each of the *pqqC* primers described in Meyer et al. [6] (forward pqqCf1, 5′-CAGGGCTGGGTCGCCAACC-3′ and reverse pqqCr1, 5′-CATGGCATCGAGCATGCTCC-3′), 0.75 U Taq DNA-Polymerase (5000 U/ml, New England Biolabs, Ipswich, MA, USA) and 2 µl of genomic DNA. For DGGE analysis the forward primer pqqCf1 contained a 40 bp GC-clamp at the 5′ (5′-CGCCCGCCGCGCCCCGCGCCCGTCCCGCCGCCCCCGCCCG-3′). The following thermocycling conditions were used: initial denaturation at 96°C for 10 min followed by 30 (or 35) cycles of 96°C for 30 s, 63°C for 30 s, 72°C for 1 min and final elongation at 72°C for 10 min. For *pqqC* amplification from roots, 20 ng total DNA extracts, 5% dimethylsulfoxide (Sigma-Aldrich, St. Louis, MO, USA) and 5% bovine serum albumin were added to the PCR mix. The presence of amplified fragments was checked by standard gel electrophoresis and ethidium bromide staining.

**DGGE analysis** was performed using the DCode Universal Mutation Detection System (Bio-Rad, Hercules, CA, USA). DGGE gels for both types of PCR products, i.e. amplified from cell lysates and DNA extracts from roots, were cast using a double gradient ranging from 7 to 12% acrylamide and from 40 to 60% denaturant (100% denaturant corresponding to 7 M urea and 40% deionized formamide). The samples were run for 13–14 h at 150 V in 1× TAE buffer (40 mM Tris base, 20 mM acetic acid, 1 mM EDTA, pH 8) preheated at 60°C. The gels were stained with SYBR Gold (Molecular Probes, Eugene, OR, USA) for 1 h and visualized with a UV trans-illuminator. In order to test the suitability of this method for determination of the diversity of root colonizing pseudomonads, a set of reference pseudomonads (Table S1) was analyzed by *pqqC*-DGGE: 10 *Pseudomonas* isolates from five different species (*P. chlororaphis*, *P. aeruginosa*, *P. corrugata*, *P. fluorescens* and *P. putida*), 19 biocontrol DAPG-producing *Pseudomonas* sp. strains belonging to different multilocus groups as defined by Frapolli et al. [61], and 31 wheat root isolates (RW09-C35 to C65) from the Reckenholz field trial 2008. The DGGE standard marker consisted of *pqqC* fragments generated from *P. fluorescens* Q2-87, *P. fluorescens* CHA0, *P. chlororaphis* 1245, *P. fluorescens* S8-151, *P. fluorescens* K94.37, and *Pseudomonas* sp. RW09-C35 which were mixed after PCR amplification at equal concentrations.

#### Sequencing and cloning of DGGE bands

DGGE bands were characterized as described previously [36]. Briefly, the central part of DGGE bands was cut out using sterile pipette tips. The gel pieces were then washed with 100 µl sterile bidistilled H_2_O at room temperature for 1 h and used as template for a 40 µl PCR reaction with primers pqqCf1 and pqqCr1 with the conditions described above. Each main band used for diversity analysis was sequenced or cloned from different wheat root samples. Cloning was performed using the TA cloning vector pJET1.2 (CloneJet PCR cloning kit; Fermentas, Glen Burnie, MD, USA). The constructs were transformed into chemically competent *Escherichia coli* One Shot® TOP 10 cells (Invitrogen, Carlsbad, CA, USA), and transformants containing the pJET1.2_*pqqC* construct were selected for sequencing.

#### Phylogeny of DGGE bands

A phylogenetic tree was inferred from the *pqqC* sequences (501 bp) of (i) the DGGE bands, (ii) a selection of 14 reference pseudomonas including 11 *Pseudomonas* strains listed in Table S1 and three GenBank sequences (*P. fluorescens* SBW25, GenBank database accession number NC_012660.1; *P. fluorescens* Pf0-1, NC_007492.2; and *P. syringae* pv. *syringae* B728a, CP000075.1), (iii) 30 RW09-C isolates (Table S1), and (iv) 76 RW09-NC clones obtained from wheat roots as described in Meyer et al. [6] (Genbank database accession numbers JN397402 - 477). The alignment of DNA sequences was performed with ClustalW 1.8 implemented in the MEGA software version 4.0 package [62]. The phylogenetic tree shown in Fig. S1 was constructed with the MEGA software version 4.0 [62], using the neighbour-joining (NJ) method [63].

#### Analysis of DGGE band patterns

DGGE patterns were converted into binary matrices (presence vs. absence of bands for each plant replicate) and subsequently the results of four or five (Reckenholz 2009) replicates were converted to frequency matrices (frequency of band occurrence). Correspondence analysis (CA) was performed on frequency matrixes using the Vegan package in R Project for Statistical Computing, version 2.11.1 [64]. Multidimensional scaling (MDS) was built to analyse relationship among samples based on the binary matrices. MDS was performed with R, using the Jaccard coefficient. MRPP (Multiple Response Permutation Procedure) [65] was used to examine potential impacts of plant production procedure, plant age and sampling year on the diversity of the *Pseudomonas* communities on wheat roots, based on their DGGE profiles (presence/absence matrices of individual replicates and frequency matrices of pooled replicates). MRPP was performed in R, using 999 permutation runs and the Jaccard distance matrix. In MRPP a significant delta statistic (P<0.05) indicates that the groups are more different than expected by chance; however it is dependent on the sample size. The MRPP calculates also an effect size A (chance-corrected within-group agreement), which relates the observed intra-group average distance to the mean of the calculated distribution. When the effect size A = 1, all items within groups are are identical. The closer A is to 1, the tighter is the grouping. In ecology A>0.3 is fairly high and A<0.1 is common.

Additionally, to analyze relationships between diversity based genotype numberand factors such as plant line, replicate (block) effect, plant age, field season, plant production procedures and damage level (vandals damage) a generalized linear model (glm) [66] was fitted on the number of *pqqC* genotypes/bands present per plant sample using R.

### Statistical analysis of population sizes

*Pseudomonas* spp. and AMF population sizes were analyzed by analysis of variance (ANOVA) with a significant threshold below 0.05 using the software GenStat 13^th^ Edition (VSN International Ltd). *Pseudomonas* population size data were log-transformed prior to the analysis. Then multiple regression models were fitted to analyze the effects of different factors which varied between individual field trials. The normality and homoscedasticity of the model was examined through residual plots. Models with three hierarchical steps were used for the analysis of Bobwhite lines: 1) comparing the Bowhite parental line with all manipulated lines (GM and sister lines), 2) comparing all GM lines with all sister lines and 3) comparing individual GM or sister lines (e.g. Pm3b#1 vs Pm3b#2) or 1) comparing Bobwhite parental line with all manipulated lines 2) comparing lines derived from individual transformation events (Pm3b#1 and Sb#1 vs Pm3b#2 and Sb#2 and 3) comparing individual sister lines and GM lines (e.g. Pm3b#1 vs. Sb#1).

On 13^th^ June 2008 (between the first and the second sampling) part of the plots were destroyed by an act of vandalism. To estimate this impact, plant damage was scored in each plot. A regression analysis showed that total *Pseudomonas* population sizes on roots harvested at the milky ripe stage diminished slightly with increasing plot damage: Y (log CFU/g roots) = −0.25×(plot damage level)+7.80, R^2^ = 0.12, n = 320 (number of plots of all plants present on the field) and mycorrhizal population increased slightly: Y (% colonized roots) = 0.015×(plot damage level)+0.09, R^2^ = 0.06, n = 320. This damage effect could be removed in the graphical representations by multiplying the data with the slope from the regression analysis and in the statistical analysis by adding the factor plot damage as a covariance. Error bars displayed in the figures represent the root mean square error (also known as the standard error of the estimate).

## Supporting Information

Figure S1 **Phylogenetic relationship among** ***pqqC*** **-DGGE bands obtained from wheat root samples in the Reckenholz field trials 2008 and 2009,** ***Pseudomonas*** **reference strains and** ***Pseudomonas*** **wheat root isolates.**
(DOC)

Figure S2 **Multi-dimensional scaling (MDS) plots of** ***pqqC*** **-DGGE profiles from wheat root samples.**
(DOC)

Table S1 **Bacterial strains and isolates used in this study.**
(DOC)

Table S2 **Significant factors impacting on** ***pqqC*** **diversity within pseudomonads colonizing wheat roots in the field trials performed at Reckenholz in 2008 (experiment 1,** **Table 1** **) and 2009.**
(DOC)
