# Supplementary material for: Does Wheat Genetically Modified for Disease Resistance Affect Root-Colonizing Pseudomonads and Arbuscular Mycorrhizal Fungi?
Source: PLoS One. 2013 Jan 23;8(1):e53825. doi: 10.1371/journal.pone.0053825 (PMC3553117; doi:10.1371/journal.pone.0053825)
Supplement: Figure S1 — Phylogenetic relationship among pqqC -DGGE bands obtained from wheat root samples in the Reckenholz field trials 2008 and 2009, Pseudomonas reference strains and Pseudomonas wheat root isolates. (DOC) [file pone.0053825.s001.doc]

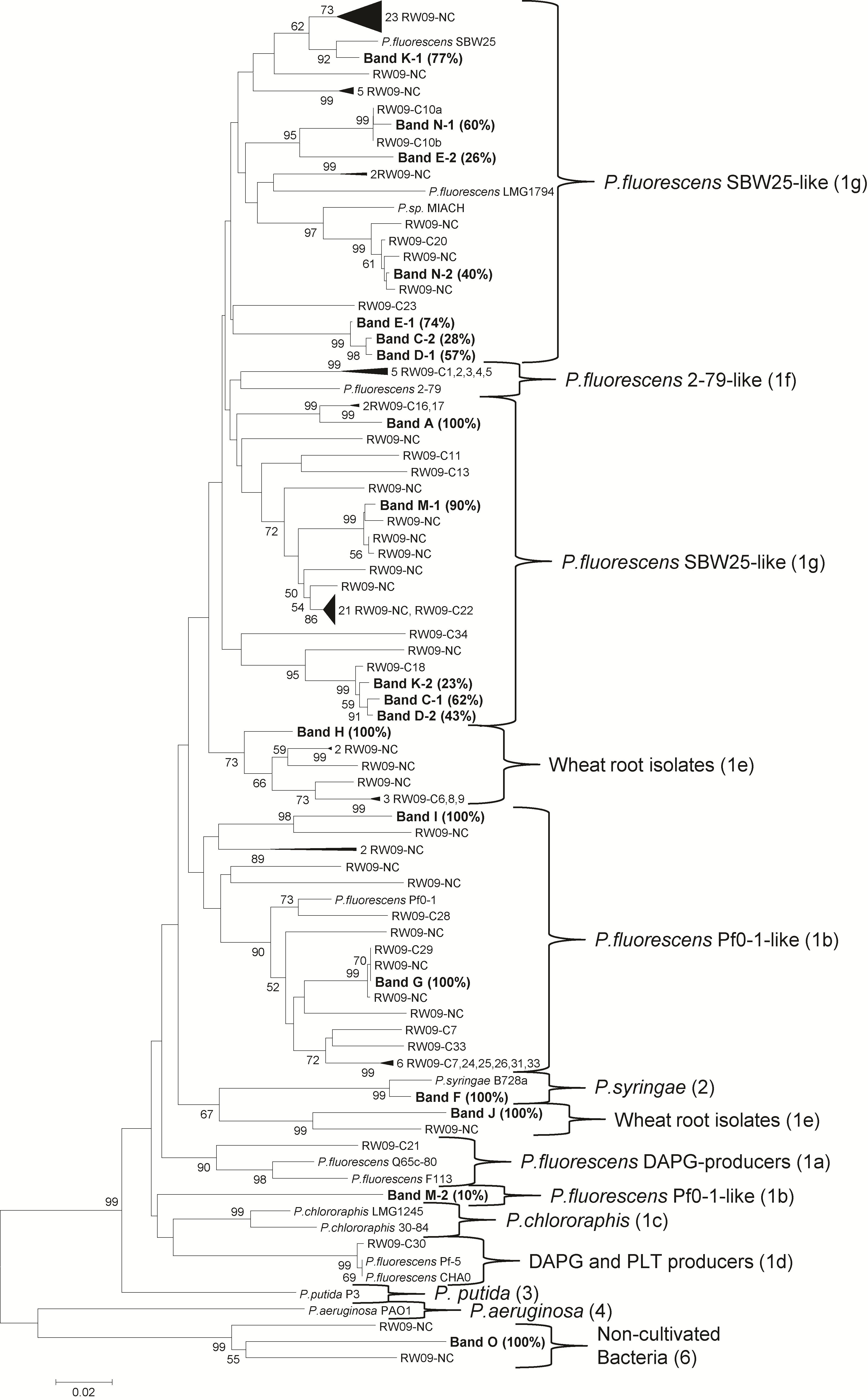


**Figure S1.** **Phylogenetic relationship among *pqqC*-DGGE bands obtained from wheat root samples** **in the Reckenholz field trials 2008 and 2009, *Pseudomonas* reference strains and *Pseudomonas* wheat root isolates.** The neighbour-joining (NJ) tree was inferred from *pqqC* (501 bp) sequences. Only bootstrap values greater than 50% are shown. Scale bar = 0.02 substitutions per site. RW09x = *pqqC* sequences from *Pseudomonas* isolates, RW09-NC = cloned *pqqC* sequences, both obtained from Bobwhite grown in the Reckenholz field trial 2009 [6]. The percentage in brackets behind a band designation indicates the fraction of analyzed bands sharing the same motility in the gel (designated with the same letter and with -1 or -2) also sharing the same *pqqC* sequence, eg bands N1 and N2 share the same motility but not the same sequence.
