# Supplementary material for: Does Wheat Genetically Modified for Disease Resistance Affect Root-Colonizing Pseudomonads and Arbuscular Mycorrhizal Fungi?
Source: PLoS One. 2013 Jan 23;8(1):e53825. doi: 10.1371/journal.pone.0053825 (PMC3553117; doi:10.1371/journal.pone.0053825)
Supplement: Figure S2 — Multi-dimensional scaling (MDS) plots of pqqC -DGGE profiles from wheat root samples. (DOC) [file pone.0053825.s002.doc]

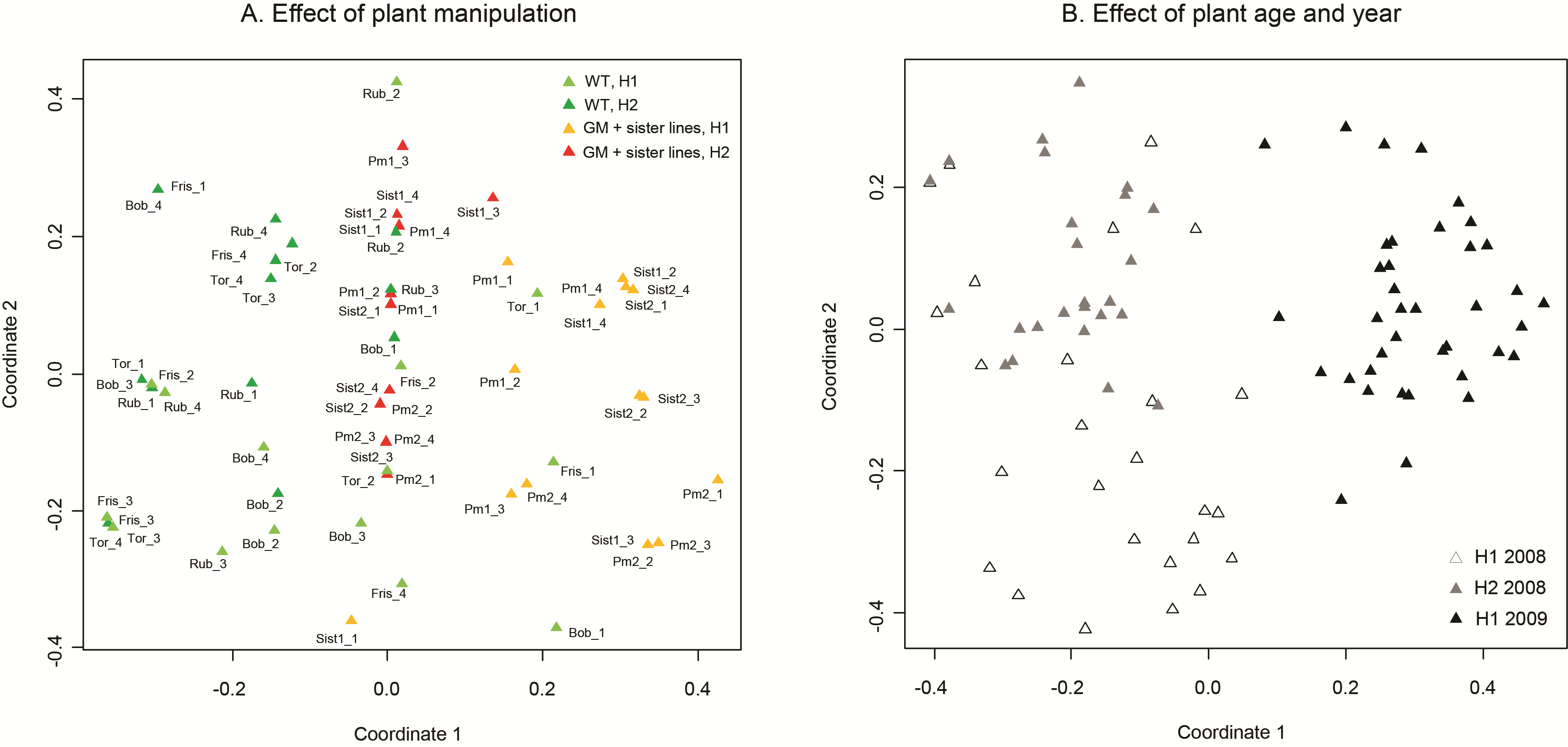


**Figure S2. Multi-dimensional scaling (MDS) plots of *pqqC-*DGGE profiles from wheat root samples.** Graph A shows plants grown in the Reckenholz field trial in 2008 (experiment 1) and sampled at the tillering (H1) and at the milky ripe stage (H2). Graph B additionally includes data from Reckenholz field trial 2009 obtained from plants harvested at the tillering stage (H1). GM and SIST = GM Bobwhite (*pm3b*) and non-GM Bobwhite sister lines; WT = conventional non-GM wheat cultivars Frisal (or Fris), Toronit (or Tor), Rubli (or Rub) and Bobwhite (or Bob).
