# Supplementary material for: Does Wheat Genetically Modified for Disease Resistance Affect Root-Colonizing Pseudomonads and Arbuscular Mycorrhizal Fungi?
Source: PLoS One. 2013 Jan 23;8(1):e53825. doi: 10.1371/journal.pone.0053825 (PMC3553117; doi:10.1371/journal.pone.0053825)
Supplement: Table S1 — Bacterial strains and isolates used in this study. (DOC) [file pone.0053825.s003.doc]

**Table S1.** Bacterial strains and isolates used in this study

| Bacterial strains | Comments | | Reference |
| --- | --- | --- | --- |
| **Reference pseudomonads** ^a^ | | | |
| DAPG-producing *P. fluorescens* strains: F113, Q65c-80, S8-151, TM1B2, K94.37, C10-204, Q37-87, Q2-87, Q13-87, Q86-87, C10-186, K93.52, PILH1, F96.26, P97.38, F96.27, CHA0, Pf-5, S8-62 | Biocontrol strains | [61] | |
| *P. chlororaphis* 30-84 | Biocontrol strain | [67] | |
| *P. chlororaphis* LMG1245 ^T^ | Type strain | BCCM ^b^ | |
| *P. chlororaphis* LMG5004 ^T^ | Type strain | BCCM | |
| *P. corrugata* LMG2172 ^T^ | Plant pathogen, type strain | BCCM | |
| *P. fluorescens* 2-79 | Biocontrol strain | [68] | |
| *P. fluorescens* LMG1794 ^T^ | Type strain | BCCM | |
| *P. fluorescens* MIACH | Soil bacterium | [6] | |
| *P. putida* LMG2257 ^T^ | Type strain | BCCM | |
| *P. putida* P3 | Soil bacterium | UNIL ^b^ | |
| *P. aeruginosa* PAO1 | Laboratory strain | [69] | |
| ***Pseudomonas* wheat root isolates** | | | |
| 34 isolates designated RW09-C1 to RW09-C34 | Rhizosphere isolates | [6] | |
| 31 isolates designated RW09-C35 to RW09-C65 | Rhizosphere isolates | This study | |

^a^ Underlined strains were included in the phylogenetic analysis presented in Fig. S1 (supplemental material).

^b^ Abbreviations. BCCM: Belgian Co-ordinated Collections of Microorganisms. UNIL = University of Lausanne.

# References

6. Meyer JB, Frapolli M, Keel C, Maurhofer M (2011) Pyrroloquinoline quinone biosynthetic gene *pqqC*: a novel molecular marker for studying phylogeny and diversity of phosphate-solubilizing pseudomonads. Appl Environ Microbiol 77: 7345-7354.

61. Frapolli M, Défago G, Moënne-Loccoz Y (2007) Multilocus sequence analysis of biocontrol fluorescent *Pseudomonas* spp. producing the antifungal compound 2,4-diacetylphloroglucinol. Environ Microbiol **9:** 1939-1955.

67. Pierson LS III, Thomashow LS (1992) Cloning and heterologous expression of the phenazine biosynthetic locus from *Pseudomonas aureofaciens* 30-84. Mol Plant Microbe Interact 5: 330-339.

68. Weller DM, Cook RJ (1983) Suppression of take-all of wheat by seed treatment with fluorescent pseudomonads. Phytopathology 73: 463-469.

69. Holloway BW (1955) Genetic recombination in *Pseudomonas aeruginosa*. J Gen Microbiol 13: 572-581.
