# Supplementary material for: Does Wheat Genetically Modified for Disease Resistance Affect Root-Colonizing Pseudomonads and Arbuscular Mycorrhizal Fungi?
Source: PLoS One. 2013 Jan 23;8(1):e53825. doi: 10.1371/journal.pone.0053825 (PMC3553117; doi:10.1371/journal.pone.0053825)
Supplement: Table S2 — Significant factors impacting on pqqC diversity within pseudomonads colonizing wheat roots in the field trials performed at Reckenholz in 2008 (experiment 1, Table 1 ) and 2009. (DOC) [file pone.0053825.s004.doc]

**Table S2**. Significant factors impacting on *pqqC* diversity within pseudomonads colonizing wheat roots in the field trials performed at Reckenholz in 2008 (experiment 1, Table 1) and 2009.

| A. PLANT LINE/CULTIVAR EFFECT | Estimate^a^ | Signif. code^a^ |
| --- | --- | --- |
| 1.Overall model (2008) ^b^ | 1.43 | ≤ 0.001* |
| Factors ^c^: |  |  |
| Line Pmb3b#1 | 0.39 | ≤ 0.10 |
| Line Sb#1 | 0.34 | ≤ 0.10 |
| Plant age: milky ripe stage | 0.28 | ≤ 0.01* |
| 2.Overall model (2009) ^b^ | 2.22 | ≤ 0.001* |
| Replicate block | 0.31 | ≤ 0.10 |
| 3.Overall model (2008-2009) ^b^ | 1.53 | ≤ 0.001* |
| Factors ^c^: |  |  |
| Plant age: milky ripe stage | 0.28 | ≤ 0.01* |
| Field season 2009 | 0.38 | ≤ 0.05* |
| B. PLANT PRODUCTION PROCEDURES EFFECT | Estimate | Signif. code |
| 1.Overall model (2008) ^b^ | 1.69 | ≤ 0.001* |
| Factors ^c^: |  |  |
| Plant production procedure: conventional cultivars | -0.19 | ≤ 0.10 |
| Plant age: milky ripe stage | 0.28 | ≤ 0.01* |
| 2.Overall model (2009) ^b^ | 2.09 | ≤ 0.001* |
| Replicate block | -0.31 | ≤ 0.10 |
| 3.Overall model (2008-2009) ^b^ | 1.61 | ≤ 0.001* |
| Factors ^c^: |  |  |
| Plant age: milky ripe stage | 0.28 | ≤ 0.01* |
| Field season 2009 | 0.38 | ≤ 0.05* |

^a^ Estimate = regression coefficient of each term, Signif. code = the significance code for the p-values.

^b^ General linear model (glm) analysis was performed on the total number of *pqqC*-DGGE bands/genotypes per plant replicate. Overall model (2008) was performed with data of the Reckenholz field trial 2008 (experiment 1) derived from young and old plants (number of samples = 64). Overall model (2009) was performed with data of the Reckenholz field trial 2009 derived from young plants (number of samples= 40). Overall model (2008-2009) was performed with data of both trials together 2008 and 2009 (number of samples = 104).

^c^ Factors included in the glm model. A: Line (individual plant lines and cultivars), B: plant production procedures (*Pm3b* and sister lines vs all conventional cultivars), A and B: plant age (tillering vs milky ripe stage), replicate block, field year (2008 and 2009).
